# Blood Viscosity in Subjects With Type 2 Diabetes Mellitus: Roles of Hyperglycemia and Elevated Plasma Fibrinogen

**DOI:** 10.3389/fphys.2022.827428

**Published:** 2022-02-25

**Authors:** Jiehui Sun, Keqin Han, Miao Xu, Lujuan Li, Jin Qian, Li Li, Xuejin Li

**Affiliations:** ^1^Department of Endocrinology and Metabolism, Ningbo First Hospital, Ningbo, China; ^2^Department of Engineering Mechanics, Zhejiang University, Hangzhou, China

**Keywords:** red blood cell, blood flow, blood viscosity, diabetes mellilus, RBC aggregation, RBC deformation

## Abstract

The viscosity of blood is an indicator in the understanding and treatment of disease. An elevated blood viscosity has been demonstrated in patients with Type 2 Diabetes Mellitus (T2DM), which might represent a risk factor for cardiovascular complications. However, the roles of glycated hemoglobin (HbA_1c_) and plasma fibrinogen levels on the elevated blood viscosity in subjects with T2DM at different chronic glycemic conditions are still not clear. Here, we evaluate the relationship between the blood viscosity and HbA_1c_ as well as plasma fibrinogen levels in patients with T2DM. The experimental data show that the mean values of the T2DM blood viscosity are higher in groups with higher HbA_1c_ levels, but the correlation between the T2DM blood viscosity and the HbA_1c_ level is not obvious. Instead, when we investigate the influence of plasma fibrinogen level on the blood viscosity in T2DM subjects, we find that the T2DM blood viscosity is significantly and positively correlated with the plasma fibrinogen level. Further, to probe the combined effects of multiple factors (including the HbA_1c_ and plasma fibrinogen levels) on the altered blood viscosity in T2DM, we regroup the experimental data based on the T2DM blood viscosity values at both the low and high shear rates, and our results suggest that the influence of the elevated HbA_1c_ level on blood viscosity is quite limited, although it is an important indicator of glycemic control in T2DM patients. Instead, the elevated blood hematocrit, the enhanced red blood cell (RBC) aggregation induced by the increased plasma fibrinogen level, and the reduced RBC deformation play key roles in the determination of blood viscosity in T2DM. Together, these experimental results are helpful in identifying the key determinants for the altered T2DM blood viscosity, which can be used in future studies of the hemorheological disturbances of T2DM patients.

## 1. Introduction

Blood is a non-Newtonian fluid that delivers necessary substances—such as nutrients and oxygen—to living cells and removes metabolic waste products (Popel and Johnson, 2005; Secomb, 2017). In vertebrates, it is composed primarily of blood cells suspended in blood plasma. In microcirculation, blood cells are subjected to intense mechanical stimulation from both blood flow and vessel walls; hence, their mechanical and rheological properties are important to their effectiveness in performing their biological functions (Yedgar et al., 2002; Baskurt and Meiselman, 2003; Yao et al., 2003; Chen et al., 2007; Fedosov et al., 2014a; Barshtein et al., 2016, 2018, 2021; Sohrabi et al., 2017; Tan et al., 2018; Xiao et al., 2020; Chien et al., 2021). A change in blood rheological property is usually linked to blood disorders; therefore, understanding the flow dynamics and rheological properties of blood allows us to know how blood viscosity impacts cognitive functions, and provides direction for therapeutic interventions (Chien et al., 1970a; Ballas et al., 1989; Fedosov et al., 2014b; Du et al., 2015; Barshtein et al., 2017; Perakakis et al., 2018). Blood viscosity has been extensively investigated and now it is generally believed that five factors, namely blood hematocrit (Hct), red blood cell (RBC) deformability, RBC aggregation, plasma viscosity, and temperature, primarily determine the rheological behavior of blood (Chien et al., 1966, 1967, 1970b; Berger and King, 1980; Barshtein et al., 2007; Fedosov et al., 2011b; Lei and Karniadakis, 2013). In blood flow, the RBC aggregation is attributed to the macromolecules such as plasma protein fibrinogen and synthetic polymer dextran, which promote the formation of RBC aggregates in the form of rouleaux (Brust et al., 2014; Krüger-Genge et al., 2019) and eventually lead to an increased blood viscosity (Matsuda and Murakami, 1976; Tomaiuolo et al., 2016). The deformability of the RBCs, or the ability of the RBCs to deform their shape under applied stress, plays an important role in the main function of the RBCs. In pathological conditions, these alterations could result in impaired blood flow and other aspects of vascular complications. For example, the malaria-infected RBCs show progressing alteration of their mechanical and adhesive properties as the parasite develops (Zhang et al., 2015; Dearnley et al., 2016; Banas et al., 2021). These changes greatly affect rheological properties of malaria-infected RBCs and lead to obstructions of small capillaries (Shelby et al., 2003; Fedosov et al., 2011a).

Diabetes mellitus (DM), the fastest growing chronic disease worldwide, is a metabolic disease characterized by persistently elevated glucose levels in the blood (Mathers and Loncar, 2006; Deng et al., 2021). Generally, the most widely used indicators of glycemic control for diabetic patients are the blood glucose level and the glycated haemoglobin (HbA_1c_) level. Compared to the former, the HbA_1c_ level has little biological variability and reflects the average glucose concentration over the preceding 8–12 weeks (Sacks, 2011). In addition, the glycated serum protein (GSP) level, which is the amount of glucose attached to total serum proteins, offers an alternative approach for assessing glycemia in instances where HbA_1c_ may be of limited value such as pregnancy and reduced RBC lifespan. Type 2 diabetes mellitus (T2DM), the most common type of DM, is characterized by relative insulin deficiency caused by pancreatic cell dysfunction and insulin resistance in target organs (Chatterjee et al., 2017). Individuals with T2DM usually suffer from elevated level of HbA_1c_, which has been identified as an emerging risk factor for developing microvascular and macrovascular complications, (Fowler, 2008) such as diabetic retinopathy, (Brazionis et al., 2008; Cho, 2011; Li et al., 2020) diabetic nephropathy, (Young et al., 1993; Davies et al., 2006; Jeganathan et al., 2008) diabetic peripheral and autonomic neuropathy (Yang et al., 2020).

The viscosity of blood is a direct measure of the resistance of blood to flow through blood vessel. According to Poiseuille's law, the rate of blood flow through a small blood vessel can be calculated from the following algebraic equation: (Phillips et al., 2008).


(1) $$\begin{array}{r} Q=\left(\pi \times r^{4}\times \Delta P\right)/\left(8\times \eta_{\text{BV}}\times L\right) \end{array}$$

where *r* and *L* are the radius and length of the blood vessel, Δ*P* is the difference in blood pressure between the ends of the blood vessel, and η_BV_ is the viscosity of blood. Holding other parameters constant, higher blood viscosity should retard blood flow through blood vessels, which would contribute to insulin resistance and T2DM and eventually lead to diabetic microangiopathy and other circulation problems (Fowler, 2008).

It is generally accepted that the blood viscosity is higher in T2DM patients than in non-diabetic control subjects (Skovborg et al., 1966; Turczynski et al., 2003; Tamariz et al., 2008). Although the reasons for the elevation in blood viscosity are still under investigation, it is believed that the osmotic diuresis, consequence of high HbA_1c_ level, could contribute to increase blood hematocrit and reduce plasma volume (Agrawal et al., 2016). It has also been suggested that the reduced RBC deformability, increased RBC and platelet aggregation, and enhanced platelet adhesion to activated endothelium would contribute to blood hyperviscosity (Schmid-Schönbein and Volger, 1976; McMillan, 1983; Beamer et al., 1997; Cho et al., 2008; Cloutier et al., 2008; Chang et al., 2017, 2018; Li et al., 2018). For example, Turczynski et al. (2003) showed that the T2DM blood viscosity is positively correlated with retinopathy severity, which is attributed to the decreased RBC deformability. Skovborg et al. (1966) revealed that the blood viscosity of diabetic subjects is around 20% higher than that of controls. Ercan et al. (2002) suggested that the elevated plasma cholesterol contributes to increased blood viscosity by an additional effect of hyperglycemia in T2DM patients. Yazdani et al. (2021) integrated blood cell mechanics, platelet adhesive dynamics, and coagulation cascade to model the thrombus formation in diabetic blood. They showed that both the pathological alterations in the biomechanical properties of blood cells and changes in the amount of coagulation factors would contribute to the enhanced platelet adhesion and aggregation in diabetic blood. In addition, it shows that the aggregation of the RBCs is far commoner among the T2DM patients than that among the non-diabetes (MacRury et al., 1993; Babu and Singh, 2004; Deng et al., 2020). It is recognized that the plasma fibrinogen level is one of the dominant factors promoting the formation of RBC aggregates (rouleaux), which could cause an elevated blood viscosity under low shear rate (Krüger-Genge et al., 2019; Deng et al., 2020).

The viscosity of blood has long been used as an indicator in the understanding and treatment of disease (Fedosov et al., 2011a). Previous studies have examined the relationship between blood viscosity, Hct, plasma viscosity, RBC deformability, and platelet adhesion in T2DM patients. However, all studies so far have investigated the effects of just a few of the aforementioned factors in hemorheology and vascular occlusion in certain glucose conditions and for certain time. The roles of hyperglycemia and elevated plasma fibrinogen level on the altered blood viscosity at different chronic glycemic conditions is not completely clear. In this article, we evaluate the influences of both the HbA_1c_ and plasma fibrinogen levels on the T2DM blood viscosity to clarify whether the alterations in blood viscosity are appreciable in these T2DM subjects.

## 2. Materials and Methods

### 2.1. Selection of T2DM Blood Samples

In this study, blood samples from 318 patients (199 male and 119 female, mean age 56.80±12.92) with T2DM were collected during fasting glucose tests, following institutional review board (IRB) approvals from the Ningbo City First Hospital. In the fasting glucose tests, the patients were asked to fast for overnight before morning blood collection to avoid the influence of postprandial lipid increase on hemorheological characteristics (Stamos and Rosenson, 1999). To investigate the effect of the HbA_1c_ level on the hemorheological properties of T2DM blood, these blood samples were collected in a wide range of the HbA_1c_ levels. All blood samples were collected into vacuum tubes (5 ml) containing Heparin Lithium salt (75 IU/ml) anticoagulant and stored at 4 °C for *in vitro* testing within 4 h from blood withdrawal. The levels of HbA_1c_ and GSP, which are the two key parameters for the assessment of long-term glucose control in diabetes, were measured in all 318 subjects. The level of plasma fibrinogen (*c*_FN_) and other biochemical and hematologic parameters were also measured using standard methods. The values of Hct were measured as the fraction of the RBCs suspended in plasma following blood centrifugation.

The whole blood viscosity was measured at native Hct using a cone-plate viscometer (SA-6600, Beijing Succeeder Technology Inc, China). The angle between the surface of the cone and the plate was of the order of 1°, where the shear rate was regulated by rotor speed during measurement. Viscosity measurements were completed within 4 h of sample collection by the following procedures: (1) the rheometer was initialized to desired test condition (force and gap) and the turntable and dosing pin were restored to zero; (2) the temperature of the blood rheometer was pre-heated for 30 min to 37 °C; (3) the whole blood was added to the tube with a dosing needle for dilution (100:1) and mixed well; (4) the tube was placed on the pre-heated plate (37 °C); (5) the whole mensuration was then automatically controlled by the computer in a rapid, pointwise, prompt, steady-state method, and the two-dimensional curve for the blood viscosity and shear rate was traced in real-time. The test time for whole blood was within 30 s/sample.

It is known that the blood is a non-Newtonian fluid, which means that its viscosity depends on the shear rate. At low shear rates, the aggregation of RBCs induces a sharp increase in blood viscosity. At high shear rates, the blood becomes less viscous as the RBCs disaggregate, deform and align in the direction of flow. According to previous experimental and computational studies, (Skalak et al., 1981; Fedosov et al., 2011b) the blood viscosity decreased only 10% when the shear rate rises from 200.0 to 1000.0 s^-1^. In our opinion, the blood viscosity measured over a range of shear rates in 200.0 to 1.0 s^-1^ can reflect the non-Newtonian behavior of T2DM blood. Hence, in this study, the blood viscosity curves were plotted as a function of shear rate ranging from 200.0 to 1.0 s^-1^ for whole blood samples. Then, the values of whole blood viscosity (η_BV_) were chosen and analyzed at three certain shear rates (*i.e*., $\overset{∙}{\gamma }$ = 1.0 , 50.0 , and 200.0 s^-1^). The plasma viscosity (η_PV_) was measured with a capillary viscometer.

It is generally believed that surface tension plays an important role in the way liquids behave. In general, it is the property of a liquid's surface, which is caused by unbalanced forces on surface molecules that pull toward the main part of the liquid. The blood viscosity is a measure of the resistance of a liquid that is being deformed or moved. As it can be noticed that the origin of the two properties is not directly related to one another. Therefore, there is no conclusive correlation between the surface tension and viscosity (Wesołowski and Młynarczak, 2019). Hence, we did not investigate the effect of blood surface tension in the measurement of blood viscosity at low shear rates.

The ability of the RBCs to deform their shape under applied shear stress are represented by RBC deformability index (Di) or RBC rigidity index (Ri). In past decades, advances in experimental techniques (such as erythrocyte filtration, laser diffraction ellipsometry, micropipette aspiration) have allowed to measure the RBC Di and RBC Ri parameters averaged over a large number of RBCs in blood sample (Dintenfass, 1985; Nash and Meiselman, 1985; Mokken et al., 1992; Kameneva et al., 1999; Spengler et al., 2011). As suggested by previous experimental studies on the measurements of rheological characteristics of the blood, (Spengler et al., 2011) the RBC Ri parameter (i.e., the inverse of RBC deformability) is evaluated according to the following formula,


(2) $$\begin{array}{r} \text{Ri}=\frac{\eta_{\text{BV,200.0}}/\eta_{\text{PV}}-1.0}{\text{Hct}} \end{array}$$

where η_BV, 200.0_ is the whole blood viscosity at shear rate of $\overset{∙}{\gamma }$ = 200.0 s^-1^. Apparently, an increase in the RBC Ri would lead to a reduction in the RBC deformability. Additionally, the RBCs in whole blood aggregate into rouleaux at low shear rates, leading to an obvious increase in blood viscosity, whereas the large RBC aggregates can break down into small structures or isolated RBCs under high shear rates (Fedosov et al., 2011b). Herein, the RBC aggregation index (Ai) is estimated by the ratio of low shear viscosity and high shear viscosity based on the following formula, (Bull et al., 1986).


(3) $$\begin{array}{r} \text{Ai}=\frac{\eta_{\text{BV,1.0}}}{\eta_{\text{BV,200.0}}} \end{array}$$

where η_BV, 1.0_ is whole blood viscosity at shear rates of $\overset{∙}{\gamma }$ = 1.0 s^-1^.

### 2.2. Statistical Analysis

All statistical data analyses were performed using SPSS 25.0 for Windows. One-way Analysis of Variance (ANOVA) and Least Significant Difference (LSD) *post-hoc* test were used to analyze the difference in studied variables between groups with different HbA_1c_ and plasma fibrinogen levels. Pearson's correlation analysis was used to test the statistical relationship between two continuous variables. A simple correlation analysis was performed to evaluate the independent association of clinical and biochemical variables with the HbA_1c_ and plasma fibrinogen levels. In this study, the significance level was set as 0.05. If the *P*-value is lower than the significance level, it means that the relationship between two or more variables are statistically significant. Additionally, the *R*-value and confidence interval (with the confidence level of 95%) were also included in the Pearson's correlation analysis.

## 3. Results and Discussion

### 3.1. Effect of Glycated Hemoglobin Level on T2DM Blood Rheology

All T2DM blood subjects were divided into three groups according to their HbA_1c_ levels: Group A (subjects with good glycemic control, HbA_1c_ <6.5%), Group B (subjects with poor glycemic control, 6.5% ≤ HbA_1c_ < 10.0%), and Group C (subjects with the worst glycemic control, HbA_1c_ ≥ 10.0%). Selected biochemical, hematologic and hemorheological parameters in these three different groups are listed in Table 1. It shows that the levels of GSP are significantly higher in the T2DM blood subjects with higher HbA_1c_ levels (Groups B and C) compared to that in the T2DM blood subjects with lower HbA_1c_ level (Group A). The values of Hct are similar in these three different groups but the RBC counts (*N*_RBC_) are higher in Groups B and C compared to that in Group A, resulting in lower values of MCV in Groups B and C based on the following equation,


(4) $$\begin{array}{r} \text{MCV (fL)}=\frac{\text{Hct \%}}{N_{\text{RBC}}\times 10^{6}/\mu \text{L}}\times 10 \end{array}$$

The value of plasma fibrinogen level *c*_FN_ is slightly higher in Group B compared to those in other two groups; however, there are no significant differences among these three different groups (*P* = 0.079). Additionally, the values of RBC Ai and Ri are almost the same among the three groups, which indicates that these two factors remain unaffected by the changes in the HbA_1c_ level.

**Table 1:** Selected biochemical, hematologic and hemorheological characteristics of the T2DM blood subjects by tertile of the HbA_1c_ level.

**Item**	**Subjects**			***P***
	**Group A**	**Group B**	**Group C**
Sex(M/F)	40/18	96/71	63/30	**-**
Age	56.48 ± 12.92	58.82 ± 12.33	56.42 ± 13.87	0.260
Hct (%)	42.03 ± 4.23	42.86 ± 4.24	42.33 ± 4.54	0.381
MCV (fL)	92.07 ± 4.84	91.44 ± 5.25	89.74 ± 3.57	0.005
*N*_RBC_ (10^6^/μL)	4.58 ± 0.54	4.71 ± 0.58	4.73 ± 0.55	0.254
GSP (%)	2.00 ± 0.34	2.74 ± 0.63*	3.92 ± 0.832*^$^	<0.001
*c*_FN_ (mg/dL)	2.91 ± 0.43	3.07 ± 0.62	2.92 ± 0.65	0.079
η_PV_ (mPa·s)	1.38 ± 0.09	1.41 ± 0.09	1.41 ± 0.09	0.029
η_BV, 1.0_ (mPa·s)	16.70 ± 3.26	17.06 ± 2.82	16.98 ± 3.08	0.739
η_BV, 50.0_ (mPa·s)	4.45 ± 0.58	4.61 ± 0.60	4.57 ± 0.67	0.258
η_BV, 200.0_ (mPa·s)	3.80 ± 0.48	3.91 ± 0.50	3.87 ± 0.56	0.395
Ai	4.38 ± 0.63	4.37 ± 0.57	4.40 ± 0.50	0.917
Ri	3.92 ± 0.61	3.98 ± 0.58	3.92 ± 0.67	0.687

Group A: HbA_1c_ <6.5%; Group B: 6.5% ≤ HbA_1c_ <10.0%; Group C: HbA_1c_ ≥ 10.0%. Hct, hematocrit; MCV, mean cell volume; N_RBC_, RBC count; GSP, glycated serum protein; c_FN_, plasma fibrinogen level; η_PV_, plasma viscosity; η_BV, 1.0_, η_BV, 50.0_, η_BV, 200.0_ are whole blood viscosity at shear rates $\overset{∙}{\gamma }$ = 1.0 s^-1^, 50.0 s^-1^ and 200.0 s^-1^, respectively; Ai, RBC aggregation index; Ri, RBC rigidity index. *, P < 0.05, vs. Group A; ^$^, P < 0.05, vs. Group B.

With regards to the rheological properties of T2DM blood, it shows that the T2DM blood subjects with higher HbA_1c_ levels (Groups B and C) have little bit higher values of plasma viscosity η_PV_, compared to that in the T2DM blood subjects with lower HbA_1c_ level (Group A). The values of blood viscosity η_BV_ at all three selected shear rates ($\overset{∙}{\gamma }$ = 1.0, 50.0, and 200.0 s^-1^) are also higher in Groups B and C compared to those in Group A; however, there are no statistically significant differences in the mean η_BV_ values among the three groups (*P* ≥ 0.258), indicating that the HbA_1c_ level did not seem to have important effect on the blood viscosity.

**Figure 1:**
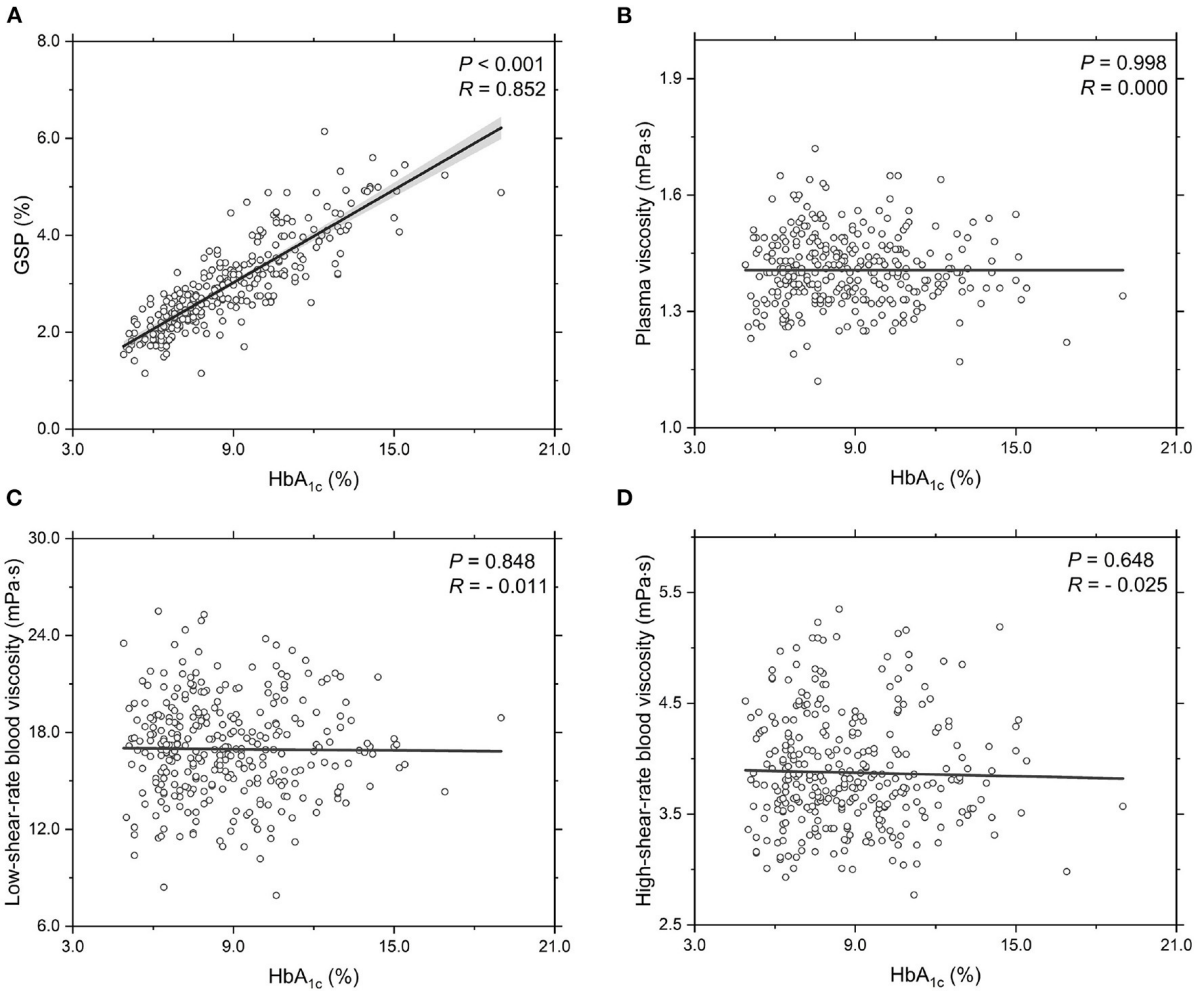
Functional dependencies of **(A)** GSP, **(B)** plasma viscosity η_PV_, **(C)** low-shear-rate blood viscosity η_BV, low_ at $\overset{∙}{\gamma }$ = 1.0 s^-1^, and **(D)** high-shear-rate blood viscosity η_BV, high_ at $\overset{∙}{\gamma }$ = 200.0 s^-1^ with respect to HbA_1c_ level. Shadow region represents 95% confidence interval.

Next, we investigate the functional dependencies of the GSP level, plasma viscosity η_PV_, low-shear-rate blood viscosity η_BV, low_ ($\overset{∙}{\gamma }$ = 1.0 s^-1^) and high-shear-rate blood viscosity η_BV, high_ ($\overset{∙}{\gamma }$ = 200.0 s^-1^) on the HbA_1c_ level, see Figure 1. It shows that the GSP level is significantly and positively correlated with the HbA_1c_ level (Figure 1A). This agrees with several reported studies (Lapolla et al., 1988; Bahceci et al., 2005). With regards to the rheological properties of T2DM blood, the measurements of plasma viscosity (Figure 1B), low-shear-rate blood viscosity (Figure 1C), and high-shear-rate blood viscosity (Figure 1D) show large fluctuations with the HbA_1c_ level. However, neither the plasma viscosity (Figure 1B) nor the blood viscosity (Figures 1C,D) shows obvious correlation with the HbA_1c_ level. These results further demonstrate that the contribution of the HbA_1c_ level to the blood viscosity seems quite limited, at least in apparently T2DM blood subjects and at the shear rates used in the present study.

### 3.2. Effect of Plasma Fibrinogen Level on T2DM Blood Rheology

In T2DM patients, the RBC aggregation induced by the plasma fibrinogen level is a key determinant of the non-Newtonian flow behavior of human blood, especially at low shear rates, which has been suggested as a possible contributing factor for the occurrence and progression of diabetic microangiopathy (Krüger-Genge et al., 2019; Deng et al., 2020; Li et al., 2020). Next, we investigate the influence of plasma fibrinogen level on the blood viscosity in T2DM.

All T2DM blood subjects are divided into three groups according to their plasma fibrinogen levels *c*_FN_: Group A (*c*_FN_ <2.5 mg/dL), Group B (2.5 mg/dL ≤ c_FN_ <3.5 mg/dL), and Group C (*c*_FN_ ≥ 3.5 mg/dL). Selected biochemical, hematologic and hemorheological parameters in these blood subjects are listed in Table 2. It shows that there are no statistically significant differences in the mean values of Hct, MCV, RBC count, and HbA_1c_ level among the three groups (*P* ≥ 0.168). Additionally, it shows that the RBC Ri remains unaffected by the changes in plasma fibrinogen level *c*_FN_. However, our results indeed show a closer correlation between the RBC Ai and the plasma fibrinogen level *c*_FN_ (*P* = 0.035), namely, the values of RBC Ai are higher in Groups B and C compared to that in Group A. These results confirm that the plasma fibrinogen mainly affects the RBC aggregation characteristics. With regards to the rheological properties of T2DM blood, it shows that the T2DM blood subjects with higher *c*_FN_ (Groups B and C) have higher values of plasma viscosity η_PV_ and blood viscosity η_BV_ at all three shear rates ($\overset{∙}{\gamma }$ = 1.0, 50.0, and 200.0 s^-1^), compared to those blood subjects with lower *c*_FN_ (Group A). For example, the values of low-shear-rate blood viscosity η_BV, low_ at $\overset{∙}{\gamma }$ = 1.0 s^-1^ in Groups B and C were around 7.6 and 8.7% higher than that in Group A.

**Table 2:** Selected biochemical, hematologic and hemorheological characteristics of the T2DM blood subjects by tertile of the plasma fibrinogen level *c*_FN_.

**Item**	**Subjects**			***P***
	**Group A**	**Group B**	**Group C**
Sex(M/F)	42/11	127/95	30/13	**-**
Age	55.11 ± 13.14	57.93 ± 12.83	59.67 ± 12.94	0.202
Hct (%)	42.22 ± 4.26	42.45 ± 4.34	43.54 ± 4.31	0.262
MCV (fL)	90.78 ± 4.31	91.26 ± 4.78	90.37 ± 5.56	0.491
*N*_RBC_ (10^6^/μL)	4.66 ± 0.51	4.67 ± 0.57	4.84 ± 0.61	0.168
HbA_1c_ (%)	8.85 ± 2.49	8.68 ± 2.38	8.84 ± 2.49	0.857
η_PV_ (mPa·s)	1.37 ± 0.10	1.40 ± 0.09*	1.45 ± 0.09*^$^	<0.001
η_BV, 1.0_ (mPa·s)	15.94 ± 2.73	17.15 ± 2.97*	17.32 ± 3.07*	0.020
η_BV, 50.0_ (mPa·s)	4.44 ± 0.63	4.57 ± 0.60	4.73 ± 0.68*	0.064
η_BV, 200.0_ (mPa·s)	3.78 ± 0.53	3.87 ± 0.49	4.04 ± 0.58*^$^	0.043
Ai	4.24 ± 0.52	4.43 ± 0.57*	4.30 ± 0.53	0.035
Ri	4.01 ± 0.55	3.93 ± 0.62	3.99 ± 0.67	0.617

Group A: c_FN_ <2.5 mg/dL; Group B: 2.5 mg/dL ≤ c_FN_ <3.5 mg/dL; Group C: c_FN_ ≥ 3.5 mg/dL. The selected parameters in this table are defined exactly the same as those in Table 1. *, P < 0.05, vs. Group A; ^$^, P < 0.05, vs. Group B.

**Figure 2:**
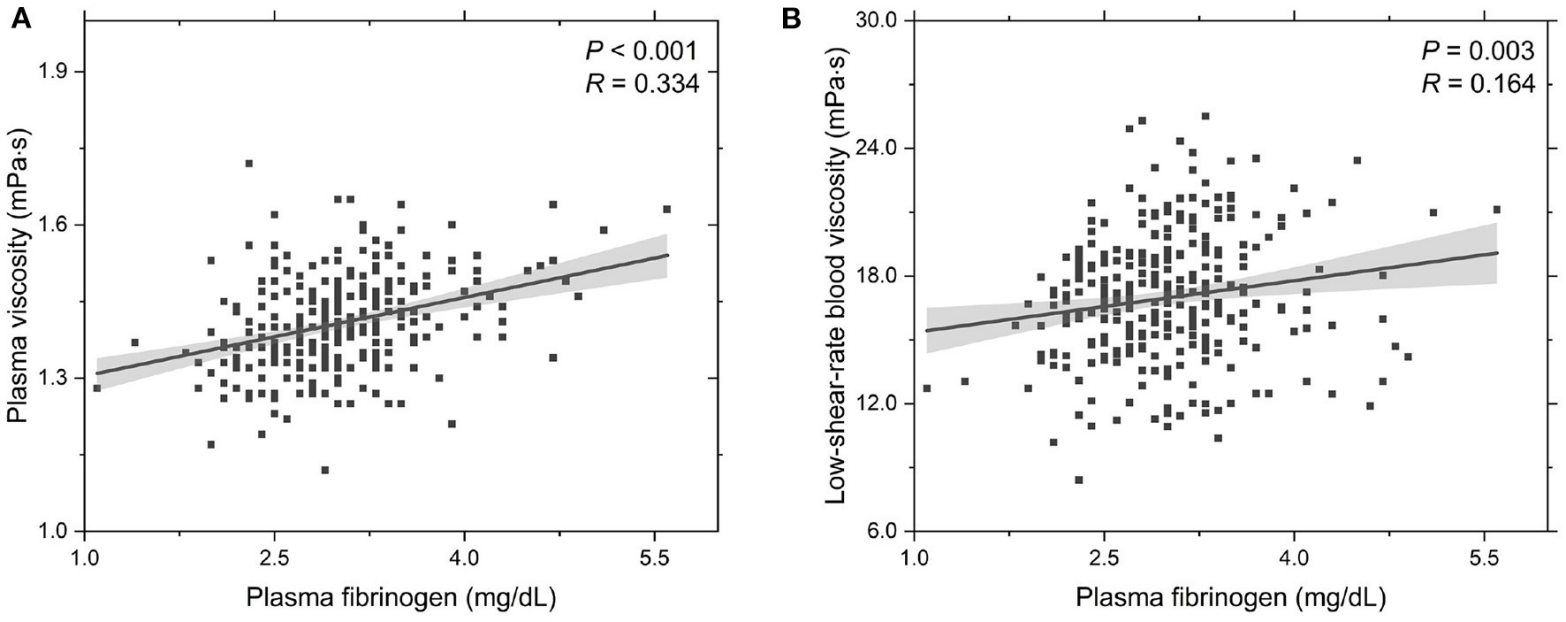
Functional dependencies of **(A)** plasma viscosity η_PV_ and **(B)** whole blood viscosity η_BV, low_ at $\overset{∙}{\gamma }$ = 1.0 s^-1^ with respect to plasma fibrinogen level *c*_FN_. Shadow region represents 95% confidence interval.

To further probe the rheological behavior of T2DM blood, we investigate the functional dependencies of plasma viscosity η_PV_ and low-shear-rate blood viscosity η_BV, low_ on the plasma fibrinogen level *c*_FN_, see Figure 2. It shows that the plasma viscosity η_PV_ positively correlated with the plasma fibrinogen level *c*_FN_ (Figure 2A), which agrees with previous experimental studies that the plasma viscosity increases with plasma fibrinogen level (Brunner, 2007). Regarding the dependence of low-shear-rate blood viscosity on plasma fibrinogen level, the values of η_BV, low_ show a positive correlation with plasma fibrinogen level (Figure 2B), which is mainly attributed to the enhanced fibrinogen-induced RBC aggregation and the increased plasma viscosity. This result is also consistent with previous experimental study that the blood viscosity elevation is associated with the increased plasma fibrinogen level (Matsuda and Murakami, 1976). Compared to the experimental results presented in Subsection 3.1, we conclude that an increased plasma fibrinogen level is more important to the elevated blood viscosity than the increase in HbA_1c_ level.

### 3.3. Multi-Factor Analysis in Altered T2DM Blood Rheology

There are several different factors affecting blood viscosity, especially under pathological conditions. Hence, an increased blood viscosity may be the reason why the other biochemical, hematologic and metabolic factors are important, and provides the underlying mechanism through which these other factors convey the pre-inflammatory insult to blood vessel walls (Sloop, 1996). An alternative method of analysis is to group patients into different categories by their blood viscosity levels (Ciuffetti et al., 2005). To probe the combined effects of multiple factors in altered blood rheology in T2DM, we consider to regroup the experimental data based on the values of blood viscosity at both the low and high shear rates. At low shear rate ($\overset{∙}{\gamma }$ = 1.0 s^-1^), all T2DM blood subjects are divided into three groups according to their blood viscosity values: Group A (η_BV, 1.0_ <15.0 mPa·s), Group B (15.0 mPa·s ≤ η_BV, 1.0_ <19.0 mPa·s), and Group C (η_BV, 1.0_ ≥ 19.0 mPa·s). Selected biochemical, hematologic and hemorheological parameters in these three blood groups are listed in Table 3. It shows that the Hct levels and RBC counts (*N*_RBC_) appear opposite trends with the age from Group A to Group C, which is in agreement with previous studies that these two variables decreased in the elderly subjects as they grow older (De Meyer et al., 2008; Zierk et al., 2020). It also shows that there are no statistically significant differences in the mean values of HbA_1c_, GSP, and RBC Ri. However, different from the results presented above, our results show that the values of Hct, plasma fibrinogen level *c*_FN_, and RBC Ai gradually increase from Group A to Group C. In a previous study by Irace et al. (2014), it has been shown that the plasma viscosity is directly associated with the low-density lipoprotein (LDL) cholesterol. Herein, we also consider the mean values of different cholesterol levels. Our results show that the values of LDL cholesterol were increased from Group A to Group C, resulting in a gradual increase in the plasma viscosity from Group A to Group C. To better understand the alteration of the T2DM blood viscosity in response to the changes in the RBC aggregation induced by the plasma fibrinogen level, we evaluate the reduced blood viscosity at low shear rate ($\overset{∙}{\gamma }$ = 1.0 s^-1^), η_rBV, low_, defined by,


(5) $$\begin{array}{r} \eta_{\text{rBV,low}}=\frac{\eta_{\text{BV,1.0}}-\eta_{\text{PV}}}{\text{Hct}} \end{array}$$

From Table 3, we find that the values of η_rBV, low_ have a remarkable increase from Group A to Group C, which is mainly due to the enhanced RBC aggregation (RBC Ai) induced by the increased level of plasma fibrinogen.

**Table 3:** Selected biochemical, hematologic and hemorheological characteristics of the T2DM blood subjects by tertile of the whole blood viscosity at low shear rate of $\overset{∙}{\gamma }$ = 1.0 s^-1^.

**Item**	**Subjects**			***P***
	**Group A**	**Group B**	**Group C**
Sex(M/F)	33/46	107/61	59/12	**-**
Age	63.64 ± 12.19	56.41 ± 12.55*	54.10 ± 12.55*	<0.001
Hct (%)	38.66 ± 3.06	42.90 ± 3.60*	46.07 ± 3.68*^$^	<0.001
MCV (fL)	92.22 ± 5.03	90.90 ± 4.49*	90.17 ± 5.11*	0.027
*N*_RBC_ (10^6^/μL)	4.20 ± 0.40	4.73 ± 0.45*	5.13 ± 0.56*^$^	<0.001
*c*_FN_ (mg/dL)	2.92 ± 0.71	2.94 ± 0.53	3.23 ± 0.58*^$^	0.001
GSP (%)	3.01 ± 0.97	2.90 ± 0.94	3.07 ± 0.92	0.434
HbA_1c_ (%)	8.94 ± 2.43	8.70 ± 2.46	8.57 ± 2.27	0.621
HDL-C (mmol/L)	1.18 ± 0.32	1.22 ± 0.33	1.16 ± 0.27	0.445
LDL-C (mmol/L)	2.90 ± 0.92	3.16 ± 0.91	3.23 ± 0.97	0.060
TG (mmol/L)	1.46 ± 0.70	1.44 ± 0.87	1.65 ± 0.80	0.155
TC (mmol/L)	4.43 ± 1.23	4.70 ± 1.23	4.79 ± 1.24	0.163
η_PV_ (mPa·s)	1.39 ± 0.09	1.40 ± 0.10	1.43 ± 0.09*^$^	0.008
Ai	3.88 ± 0.40	4.42 ± 0.42*	4.87 ± 0.54*^$^	<0.001
Ri	3.96 ± 0.64	3.97 ± 0.57	3.88 ± 0.69	0.583
η_rBV, low_ (mPa·s)	29.63 ± 3.00	34.78 ± 2.80*	40.36 ± 4.59*^$^	<0.001

Group A: η_BV, 1.0_ <15.0 mPa·; Group B: 15.0 mPa·s ≤ η_BV, 1.0_ <19.0 mPa·s; Group C: η_BV, 1.0_ >19.0 mPa·s. HDL-C, high-density lipoprotein cholesterol; LDL-C, low-density lipoprotein cholesterol; TG, triglyceride; TC, total cholesterol; η_rBV, low_, reduced blood viscosity at low shear rate. Other parameters in this table are defined exactly the same as listed in Tables 1, 2. *, P < 0.05, vs. Group A; ^$^, P < 0.05, vs. Group B.

**Figure 3:**
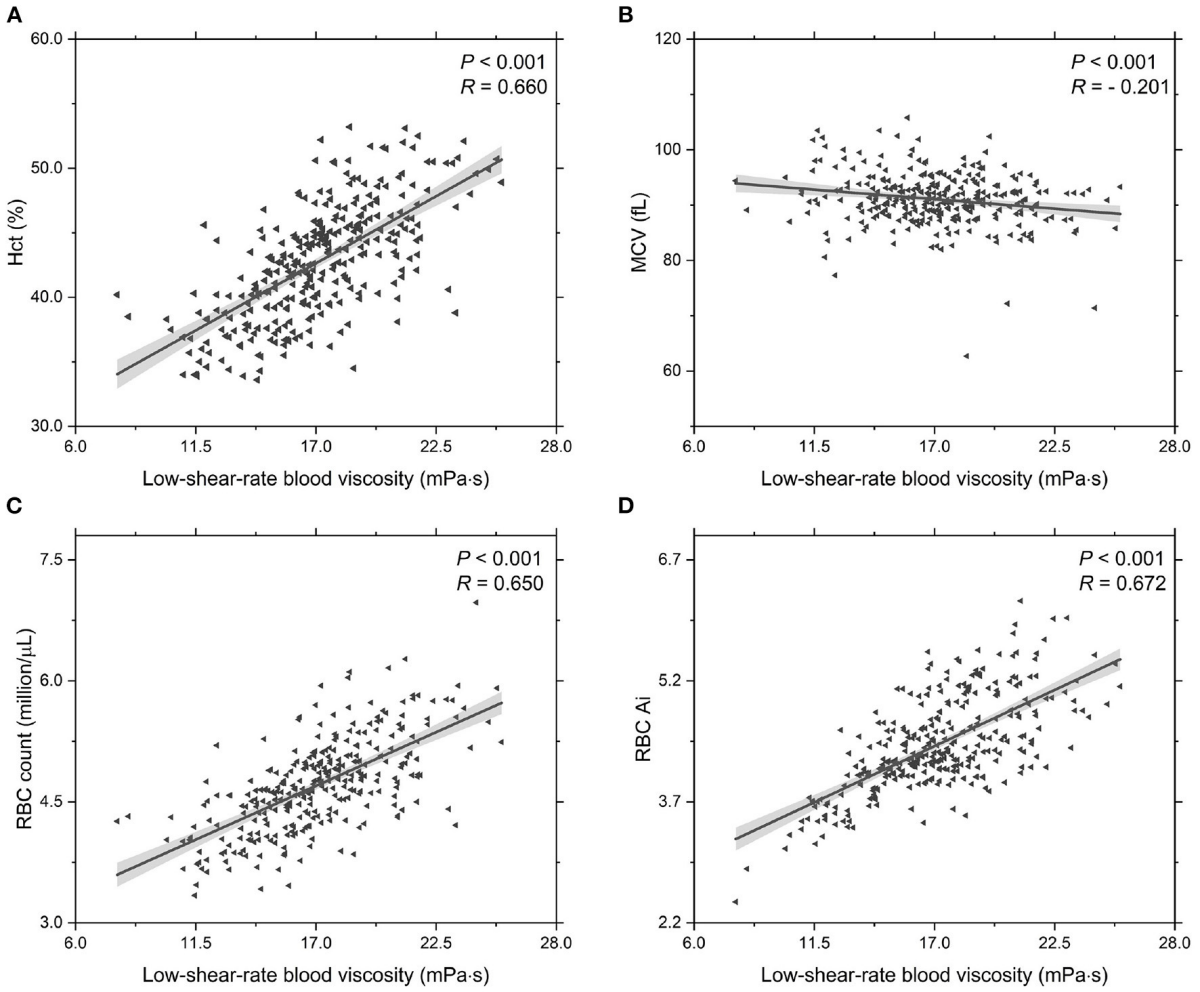
Functional dependencies of **(A)** Hct, **(B)** MCV, **(C)** RBC count, and **(D)** RBC Ai with respect to low-shear-rate T2DM blood viscosity at $\overset{∙}{\gamma }$ = 1.0 s^-1^. Shadow region represents 95% confidence interval.

In addition, we investigate the functional dependencies of Hct, MCV, RBC count and RBC Ai on the low-shear-rate blood viscosity, see Figure 3. It shows that the Hct level increases with increasing low-shear-rate blood viscosity (Figure 3A), indicating that the T2DM patients with an abnormal elevation in low-shear-rate blood viscosity have high Hct levels. From the other point of view, it confirms that the Hct level is one of the major determinants of blood viscosity. As we know, the blood hematocrit reflects the amount of space in the blood that is occupied by the RBCs, which are affected by the size of the RBCs (MCV) and by the number of RBCs (RBC counts). The results on the functional dependence of the MCV and RBC count on the low-shear-rate blood viscosity are shown in (Figures 3B,C). In general, MCV decreases (Figure 3B) while RBC counts grows (Figure 3C) with increasing low-shear-rate blood viscosity, indicating that the increased number of RBCs in the T2BM blood plays a key role in the increased Hct level, causing an elevated low-shear-rate blood viscosity. Additionally, we also probe the functional dependence of RBC Ai on the low-shear-rate blood viscosity (Figure 3D). We find that the RBC Ai is positively associated with the low-shear-rate blood viscosity, indicating that the T2DM patients with higher value of low-shear-rate blood viscosity have enhanced RBC aggregation. Take a look at it another way, we confirm that the RBC aggregation influences the low-shear-rate blood viscosity. In summary, as the values of low-shear-rate blood viscosity are gradually increased from Group A to Group C, we conclude that the T2DM blood viscosity at low shear rate is significantly associated with the increased Hct, plasma viscosity, and RBC aggregation.

At high shear rate ($\overset{∙}{\gamma }$ = 200.0 s^-1^), all T2DM blood subjects are also divided into three groups according to their blood viscosity values: Group A (η_BV, 200.0_ <3.5 mPa·s), Group B (3.5 mPa·s ≤ η_BV, 200.0_ <4.5 mPa·s), and Group C (η_BV, 200.0_ > 4.5 mPa·s). Selected biochemical, hematologic and hemorheological parameters in these three different groups are listed in Table 4. Similar to the trends obtained at low shear rate, it shows that the values of Hct, RBC count, and plasma fibrinogen level *c*_FN_ gradually increase from Group A to Group C, and the values of MCV gradually decrease from Group A to Group C. Additionally, our results show that there are no statistically significant differences in the mean values of HbA_1c_ and GSP. In contrast, we find that the values of the RBC Ri gradually increase from Group A to Group C, but the values of the RBC Ai have little changes among the three groups. Next, we evaluate the reduced blood viscosity at high shear rate, η_rBV, high_, defined by,


(6) $$\begin{array}{r} \eta_{\text{rBV,high}}=\frac{\eta_{\text{BV,200.0}}-\eta_{\text{PV}}}{\text{Hct}} \end{array}$$

From Table 4, we find that the values of η_rBV, high_ gradual increase from Group A to Group C, which could attribute to the increased RBC Ri, namely the reduced RBC deformability.

**Table 4:** Selected biochemical, hematologic and hemorheological characteristics of the T2DM blood subjects by tertile of the whole blood viscosity at high shear rate of $\overset{∙}{\gamma }$ = 200.0 s^-1^.

**Item**	**Subjects**			***P***
	**Group A**	**Group B**	**Group C**
Sex(M/F)	46/25	117/90	36/4	**-**
Age	63.15 ± 12.85	57.18 ± 12.71*	50.68 ± 10.03*^$^	<0.001
Hct (%)	38.14 ± 2.70	43.06 ± 3.41*	47.77 ± 3.62*^$^	<0.001
MCV (fL)	92.30 ± 5.27	90.88 ± 4.59*	89.80 ± 4.73*	0.021
*N*_RBC_ (10^6^/μL)	4.15 ± 0.38	4.75 ± 0.46*	5.34 ± 0.49*^$^	<0.001
*c*_FN_ (mg/dL)	2.91 ± 0.58	2.97 ± 0.57	3.29 ± 0.73*^$^	<0.01
GSP (%)	2.88 ± 1.03	2.98 ± 0.92	3.07 ± 0.91	0.590
HbA_1c_ (%)	8.91 ± 2.48	8.70 ± 2.42	8.56 ± 2.25	0.730
HDL-C (mmol/L)	1.16 ± 0.27	1.21 ± 0.34	1.14 ± 0.28	0.286
LDL-C (mmol/L)	2.89 ± 0.92	3.16 ± 0.91	3.31 ± 0.99	0.239
TG (mmol/L)	1.33 ± 0.67	1.46 ± 0.78	1.95 ± 1.05*^$^	0.036
TC (mmol/L)	4.36 ± 1.24	4.72 ± 1.21	4.85 ± 1.27	0.058
η_PV_ (mPa·s)	1.39 ± 0.10	1.40 ± 0.09	1.45 ± 0.09*^$^	<0.01
Ai	4.34 ± 0.70	4.40 ± 0.52	4.35 ± 0.49	0.658
Ri	3.41 ± 0.52	3.97 ± 0.47*	4.67 ± 0.60*^$^	<0.001
η_rBV, high_ (mPa·s)	4.70 ± 0.51	5.56 ± 0.53*	6.73 ± 0.64*^$^	<0.001

Group A: η_BV, 200.0_ <3.5 mPa·s; Group B: 3.5 mPa·s ≤ η_BV, 200.0_ <4.5 mPa·s; Group C: η_BV, 200.0_ ≥ 4.5 mPa·s; η_rBV, high_, reduced blood viscosity at high shear rate. Other selected parameters in this table are defined exactly the same as listed in Tables 1–3. *, P < 0.05, vs. Group A; ^$^, P < 0.05, vs. Group B.

**Figure 4:**
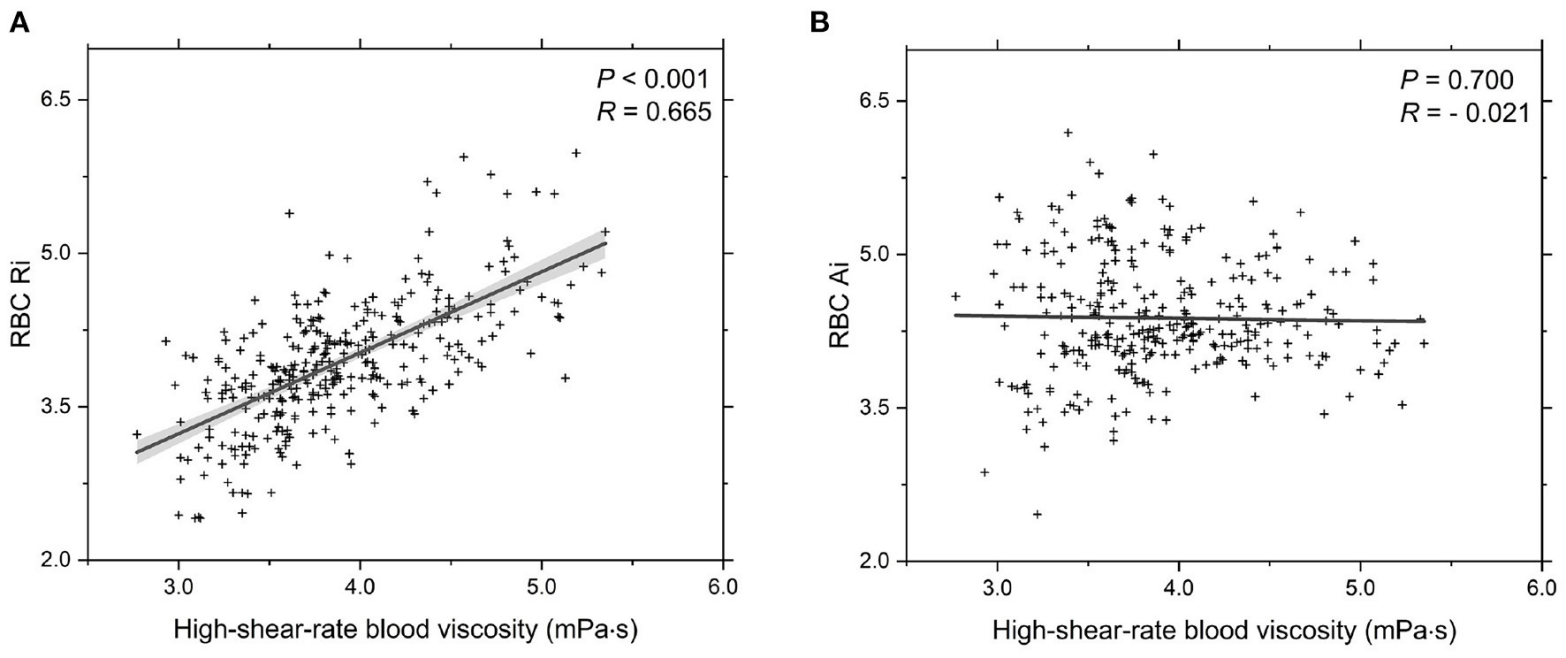
Functional dependencies of **(A)** RBC Ri and **(B)** RBC Ai with respect to high-shear-rate T2DM blood viscosity at $\overset{∙}{\gamma }$ = 200.0 s^-1^. Shadow region represents 95% confidence interval.

In addition, we investigate the functional dependencies of both the RBC Ri and RBC Ai on blood viscosity at high shear rate ($\overset{∙}{\gamma }$ = 200.0 s^-1^), see Figure 4. It shows that the values of the RBC Ri are positively correlated with the blood viscosity (Figure 4A); however, there is no correlation between the RBC Ai and the blood viscosity (Figure 4B). In fact, it is easy to understand because the formation of RBC rouleaux in blood occurs at sufficiently low shear rates. Under high shear stress conditions, the large rouleaux can break down into smaller structures or individual RBCs. Overall, these results confirm that the elevated Hct level and reduced RBC deformability are the two of the most important parameters to the elevated blood viscosity at high shear rate.

## 4. Summary

The viscosity of blood is a direct measure of the resistance of blood to flow, and an increase in blood viscosity would result in retarded blood flow thereby causing reduced delivery of substrates such as oxygen, insulin, and glucose to metabolically active tissues. In this study, we investigate the effects of glycated hemoglobin (HbA_1c_) and plasma fibrinogen levels on the rheological properties of blood in subjects with type 2 diabetes mellitus (T2DM). Our data suggest that the mean values of blood viscosity are higher in groups with higher HbA_1c_ levels; however, the correction between the blood viscosity and HbA_1c_ level is not obvious. Instead, we find that the T2DM blood viscosity is significantly and positively correlated with the plasma fibrinogen level.

Additionally, to probe the combined effects of multiple factors (including the HbA_1c_ and plasma fibrinogen levels) on the altered blood viscosity in T2DM subjects, we regroup the experimental data based on the blood viscosity values at both low and high shear rates. Our experimental results suggest that the influence of the elevated HbA_1c_ level on blood viscosity is limited, although it is an important indicator of risk for complications in T2DM patients. Instead, the increased blood hematocrit and enhanced RBC aggregation induced by the elevated plasma fibrinogen level are two of the most important parameters that determine the T2DM blood viscosity at low shear rate, and the increased blood hematocrit and reduced RBC deformation mainly contribute to the elevated T2DM blood viscosity at high shear rate.

Overall, in this study, we show that the RBC aggregation is pronounced while the RBC deformability is decreased in T2DM patients, which may cause blood flow abnormality and eventually lead to the development of vascular complications. On the one hand, the RBC hyperaggregability leads to enhanced rouleau formation at low shear rate, causing blood hyperviscosity in capillaries, reducing the delivery of substrates such as oxygen, insulin and glucose to metobolically active tissues, and eventually leading to hemodynamic impairment and vascular occlusion. On the other hand, the RBCs in patients with T2DM are associated with reduced cell deformation, which can also cause blood viscosity elevation contributing to blood flow impairment and other pathophysiological aspects of diabetes-related vascular complications such as the formation of blood clots.

## Data Availability Statement

The original contributions presented in the study are included in the article/supplementary material, further inquiries can be directed to the corresponding authors.

## Ethics Statement

The studies involving human participants were reviewed and approved by Institutional Review Board (IRB) approvals from the Ningbo First Hospital. Written informed consent for participation was not required for this study in accordance with the national legislation and the institutional requirements.

## Author Contributions

JS, KH, MX, JQ, LiL, and XL designed the research. JS and KH performed the experimental measurements and analyzed the data. All authors discussed the results and wrote this article.

## Funding

This work was supported by the Major Program of Social Development of Ningbo Science and Technology Bureau (Grant No. 2019C50094) and the Zhejiang Provincial Natural Science Foundation (Grant No. LY22A020004).

## Conflict of Interest

The authors declare that the research was conducted in the absence of any commercial or financial relationships that could be construed as a potential conflict of interest.

## Publisher's Note

All claims expressed in this article are solely those of the authors and do not necessarily represent those of their affiliated organizations, or those of the publisher, the editors and the reviewers. Any product that may be evaluated in this article, or claim that may be made by its manufacturer, is not guaranteed or endorsed by the publisher.
